# A Feasibility Study of Supply and Demand for Diabetes Prevention Programs in North Carolina

**DOI:** 10.5888/pcd14.160604

**Published:** 2017-06-29

**Authors:** Maria L. Alva, Carmen D. Samuel-Hodge, Deborah Porterfield, Tainayah Thomas, Jennifer Leeman

**Affiliations:** 1 RTI International, Washington, DC; 2 University of North Carolina at Chapel Hill, Gillings School of Global Public Health, Chapel Hill, North Carolina; 3 University of North Carolina at Chapel Hill, School of Medicine, Chapel Hill, North Carolina; 4 University of North Carolina at Chapel Hill, School of Nursing, Chapel Hill, North Carolina

## Abstract

**Introduction:**

Diabetes Prevention Programs (DPPs) have shown that healthy eating and moderate physical activity are effective ways of delaying and preventing type 2 diabetes in people with impaired glucose tolerance. We assessed willingness to pay for DPPs from the perspective of potential recipients and the cost of providing these programs from the perspective of community health centers and local health departments in North Carolina.

**Methods:**

We used contingent valuation to determine how much potential recipients would be willing to pay to participate in DPPs under 3 different models: delivered by registered professionals (traditional model), by community health workers, or online. By using information on the minimum reimbursement rate at which public health agencies would be prepared to provide the 3 models, we estimated the marginal costs per person of supplying the programs. Matching supply and demand, we estimated the degree of cost sharing between recipients and providers.

**Results:**

Potential program recipients (n = 99) were willing to pay more for programs led by registered professionals than by community health workers, and they preferred face-to-face contact to an online format. Socioeconomic status (measured by education and employment) and age played the biggest roles in determining willingness to pay. Leaders of public health agencies (n = 27) reported up to a 40% difference in the cost of providing the DPP, depending on the delivery model.

**Conclusion:**

By using willingness to pay to understand demand for DPPs and computing the provider’s marginal cost of providing these services, we can estimate cost sharing and market coverage of these services and thus compare the viability of alternate approaches to scaling up and sustaining DPPs with available resources.

## Introduction

More than 86 million Americans have prediabetes (1,2), or blood glucose levels that place them at high risk for developing diabetes. Each year approximately 5% to 10% of people with prediabetes will develop type 2 diabetes (3). Moreover, prediabetes (compared with normoglycemia) is associated with an increased risk for cardiovascular disease and all-cause mortality (3,4). However, most people with prediabetes are unaware they have it. Nationally, only about 1 in 10 people are aware they have prediabetes compared with 1 in 20 in North Carolina, where 512,000 are estimated to have diagnosed prediabetes (5).

The Diabetes Prevention Program (DPP) has shown that healthy eating and moderate physical activity are effective ways of delaying and preventing type 2 diabetes in people with impaired glucose tolerance. People at risk reduced their incidence of diabetes by 58% over 3 years (6). The DPP also saved money and reduced hospital admissions in a nonclinical setting (7). Despite evidence of the DPP’s effectiveness, this program is not yet widespread, and several barriers exist to establishing and maintaining it. The DPP’s high programmatic costs and frequency of ongoing face-to-face visits have made it challenging to implement routinely in reality (8).

As of October 2016, there were 1,074 recognized DPP providers under the Centers for Disease Control and Prevention’s (CDC’s) Diabetes Prevention Recognition Program in the United States and 40 in North Carolina (9). Expanding DPP is one of the most promising options for reducing the prevalence of diabetes. Currently, most DPPs are offered at no charge. For example, although the YMCA program costs $360 per person (10) for the duration of the core program (amounting to 16 sessions) (Appendix Table A.1), the pilot is offered at no charge. The capacity to offer free programs is limited. Further expansion of DPP will require new sources of funding. Understanding a person’s willingness to pay (WTP) is important if funding models that include member contributions are to be considered as a possible solution to the lack of prevention programs available and their potential long-term sustainability. WTP for a specified health improvement represents the maximum amount of money an individual would pay for the health improvement and still consider herself better off (11). 

To scale up the implementation of DPPs, it is important to understand community member’s WTP for these services and the costs of getting organizations to adopt DPPs. Three alternate DPP models are 1) delivered by registered professionals (traditional model), 2) delivered by community health workers (CHWs), or 3) administered online. The traditional model employs dietitians, health educators, or fitness coaches as class leaders. The CHW model employs individuals who are not registered professionals and usually work in community settings, serving as connectors between the community and health care providers. CHWs know the community culture and can relate to people better than health care professionals who may not be as locally engaged. CHWs are also less expensive than registered professionals. Online programs are an alternative to traditional models and allow people to proceed at their own pace, communicate using social media platforms, and use technology to track progress.

The purpose of this study was to measure the equilibrium WTP for alternative delivery methods and to illustrate a framework stakeholders can use to analyze the potential benefits of policies, such as subsidies, that could increase uptake.

## Methods

The study involved 2 cross-sectional surveys of potential recipients’ WTP for DPP and potential providers’ costs of delivering the program (12–18). Participants included both potential recipients and providers of DPP. Potential recipients included North Carolina adults diagnosed with prediabetes (self-reported) or identified as being at risk for prediabetes (with a score of at least 9 points on the 7-item CDC Prediabetes Screening Test) (19). A convenience sample was recruited from respondents of a previous study (20) who indicated that they would like to be contacted for future research opportunities and participant referrals of potentially eligible family members and friends. Potential providers consisted of leaders at all local health departments and community health centers in North Carolina. Providers were eligible if they were in positions of leadership in which they made decisions about prediabetes treatment services in their health agency. All study participants provided written or oral informed consent. The University of North Carolina institutional review board approved the study, and the data collection period was from April to December 2015.

### Data collection

**Consumer survey:** Trained staff administered the Consumer DPP Demand Survey by telephone to eligible adults in 16 North Carolina counties. The questionnaire consisted of 23 items covering the following categories: 1) prediabetes diagnosis and participation in weight loss programs, 2) demand or willingness to pay for DPP delivered in 3 different models, 3) preference for DPP delivery mode, 4) beliefs about DPP’s potential for delaying diabetes, and 5) demographic characteristics. To assess WTP, respondents were asked “Are you willing to pay [dollar amount] for the delivery mechanism described?” (Appendix Table A.2). Respondents who answered yes were asked about their willingness to pay for a higher amount. Respondents who answered no were offered a lower price. In our setup, respondents answered a maximum of 5 questions, for bids (in dollars) ranging from $5 to $120 per month. To minimize bias associated with the order in which respondents were asked about their WTP for the different DPP delivery models, the program sequence was randomly selected for each participant. Respondents received a $20 incentive (Wal-Mart or Target gift card) for completing the survey.

**Provider survey:** The provider survey was administered online using Qualtrics (Qualtrics). Surveys were sent to a list of 85 local health departments and 37 community health center leaders. The provider survey included items about 1) the respondent’s role, the organization, and prediabetes treatment services; 2) factors that would significantly influence respondents’ decision-making about providing DPP services; and 3) minimum price per person per month (equivalent to 4 encounters) at which they would be able to provide DPP if it was led by a registered professional (eg, dietitian, health educator, fitness coach), led by a CHW, or delivered online. To validate these answers, we also asked respondents whether they would be willing to provide DPP at varying levels of reimbursement and how many people they would enroll at these levels. Respondents received a $30 Amazon e-gift card for completing the survey.

### Analysis

**Demand valuation:** To elicit WTP using contingent valuation, we used Hanneman et al’s (21) double-bounded model maximum likelihood estimation to estimate the WTP parameters (22). Data were analyzed using Stata version 14 (StataCorp LP).

We also described how responsive (ie, elastic) the quantity demanded was to a change in price on the basis of different characteristics. We refer to this as elasticity of demand. Using the information on the minimum self-reported reimbursement rate at which potential providers would be prepared to serve their communities, we estimated the marginal costs of supplying the programs. By combining WTP estimates with marginal cost estimates of providing services, we determined the cost shares of these delivery models. Comparing uptake under different delivery models provided a measure of the extent to which outreach could be improved through CHW or online delivery (compared with traditional delivery). Elasticity of demand (E_d_) usually yields a negative value because of the inverse relationship between price and quantity demanded. To avoid confusion, we present values in absolute terms. Three demand scenarios were possible: 

*1)* *inelastic*: the percentage change in program participation is less than the percentage change in price (E_d_ < |−1|); 

*2)* *unitarily elastic*: the percentage change in participation is equal to the percentage change in price (E_d_ = |−1|); and 

*3)* *elastic*: the percentage change in participation is greater than the percentage change in price (E_d_ > |−1|).

**Supply valuation: **To estimate potential DPP providers’ supply curves (ie, marginal cost curves), we assumed constant marginal costs up to capacity. This assumption allowed respondents to ignore distinctions between fixed costs (costs incurred irrespective of the number of respondents) and variable costs (costs that change with the number of respondents enrolled). We thus assumed that the cost of providing the program does not increase with the number of people enrolled; for example, hiring a class leader or building an online program will cost the same irrespective of the class size. Once the provider reaches capacity, supply becomes perfectly inelastic; no matter what the reimbursement is, because of capacity constraints (eg, providers’ size, geography, outreach), the same number of people would be enrolled. This gives rise to a reverse-L shape with a horizontal segment (perfectly elastic supply) connected to a vertical segment (perfectly inelastic supply) at a sharp corner. Aggregating the respondents’ reverse–L supply functions gives us a representative upward sloping supply function for the state (23). For this aggregation to be representative, we assumed that each provider serves a fraction of the market. We believe this is a true approximation, because each provider in our sample serves a different county, and that distance to facilities acts as a barrier to enrollment.

### Cost sharing

Demand and supply curves are piece-wise linear by construction. The linearity around the support points allowed us to locate the intersection of demand and supply. The intersection determines prices and the fraction of the eligible population that would enroll in each program.

## Results

### Demand questionnaire

We contacted 214 potential participants; 32 did not meet the eligibility criteria, and 17 declined to participate. Of the 165 eligible participants contacted, 99 people completed the questionnaire (response rate = 60%). Respondents were predominantly female (74%), African American (60%), older than age 45 (78%), and of low and middle income, as measured by their employment status and education level (Table 1). Most (84%) respondents reported owning a computer. Approximately half of all respondents lived in Raleigh, Durham, or Kingston. Nearly 70% of respondents said there were not enough affordable weight loss programs in their community, while 91% reported living less than 20 minutes (driving time) from a gymnasium, community health center, or local health department. All respondents reported living within a 45-minute drive from one of these facilities. Lack of time, their health or that of others (caregivers), financial constraints, scheduling, and lack of motivation were the major barriers to participation in DPP. Among female respondents younger than 45, absence of child care was the major barrier (6 of 20) cited. When asked about their preferred lead for the program, most respondents selected dietitians (52%), followed by fitness coaches (23%), health educators (13%), and CHWs (11%).

**Table 1:** Characteristics of Demand Survey Participants (n = 99) and Provider Survey Participants (n = 29), Study of Supply and Demand for Diabetes Prevention Programs in North Carolina, 2015

Characteristic	Value
**Demand Survey Participants, %**	
**Female sex**	74
**Age, y**	
<45	22
45–54	20
55–64	32
≥65	26
**Race/ethnicity**	
White	36
African American	60
Hispanic	1
Other (Asian or Native American)	3
**Participated in a structured weight loss program**	39
**Participated in a Diabetes Prevention Program**	6
**Education**	
Some high school	4
High school diploma	21
Some college (13–15 years of school)	32
College degree (16 years of school)	36
Postgraduate (≥17 years of school)	6
**Has health insurance**	86
**Owns a computer**	84
**Employment status**	
Employed full-time	47
Employed part-time	7
Unemployed and seeking work	5
Retired	27
Not seeking work at the present time	13
**Provider Survey Participants, No. (%)**	
**Community health center representatives**	2 (7)
**Local health department representatives**	27 (93)
**Leadership roles**	
Health director	19 (65)
Health educator	5 (17)
Other^a^	5 (17)

a Other roles include assistant director, administrator, nurse supervisor, physician assistant, and public health officer.

**Figure 1:**
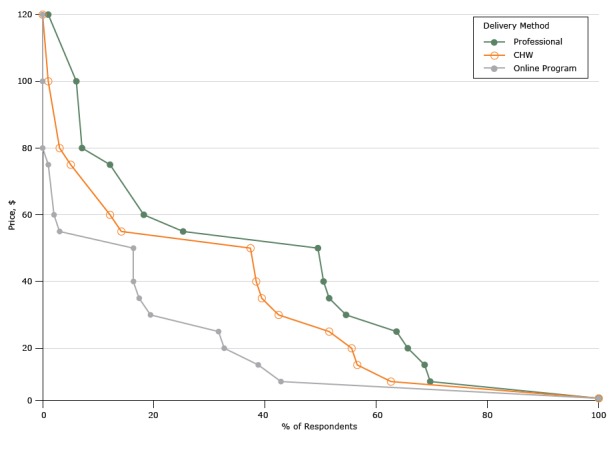
Demand for diabetes prevention programs based on willingness to pay responses (per month), North Carolina, 2015. Abbreviation: CHW, community health worker. **Table:** 

**Figure 1** shows the percentage of respondents willing to enroll in each program type at various prices per month. The unadjusted mean WTP per month was $39 (95% confidence interval [CI], $33–$44) if the DPP was led by a registered professional, $31 (95% CI, $26–$36) if led by a CHW, and $19 (95% CI, $15–$23) if administered online (Table 2). Across the 3 delivery mechanisms, people who had already participated in a structured weight loss program had a higher WTP than people who had not.

Price, $	Delivery Method, % of Respondents		
	Professional	Community Health Worker	Online Program
0	100	100	100
5	99.99	99.99	99.99
10	69.69	62.62	42.85
15	68.68	56.56	38.77
20	65.65	55.55	32.65
25	63.63	51.51	31.63
30	54.54	42.42	19.39
35	51.51	39.39	17.35
40	50.50	38.38	16.33
45	50.00	37.88	16.33
50	49.49	37.37	16.33
55	25.25	14.14	3.06
60	18.18	12.12	2.04
65	16.16	9.76	1.70
70	14.14	7.41	1.36
75	12.12	5.05	1.02
80	7.07	3.03	0
85	6.82	2.53	0
90	6.57	2.02	0
95	6.31	1.52	0
100	6.06	1.01	0
105	4.80	0.76	0
110	3.11	0.42	0
115	1.85	0.17	0
120	1.01	0	0

**Table 2:** Demand, Supply, and Subsidy Estimates, Study of Supply and Demand for Diabetes Prevention Programs in North Carolina, 2015^a^

Characteristic	Delivery Method		
	Registered Professional/Traditional	CHW	Online
**Average estimated willingness to pay**			
WTP, $ (95% CI)	39 (33–44)	31 (26–36)	19 (15–23)
**Minimum price per person per month at which local health departments or community health centers would offer DPP**			
Average price, $ (SD [min–max])	79 (85 [9–400])	49 (44 [8–200])	57 (75 [8–400])
**Cost sharing per month, assuming a $15 subsidy per person per month**			
Fraction of enrollees, %	52	50	33
Equilibrium price, $	44	31	17
Market coverage % with subsidy	57	55	46
Price to the user, $	37	25	10
Price to the provider, $	52	40	25
Cost of subsidy, $	4,377,600	4,224,000	3,532,800

Abbreviations: CHW, community health worker; CI, confidence interval; DPP, diabetes prevention program; SD, standard deviation.

a Prices are per person, per month. Twenty-seven providers answered questions across all delivery methods; we did not include answers = 0.

We also looked at WTP and demand elasticity for different sets of individual characteristics to see how responsive demand is to price (Table 3). For example, respondents who were unemployed and those who had lower levels of education (ie, did not finish high school) had a higher elasticity of demand than those who were employed and had at least a high school diploma — meaning that an increase in price would reduce their participation more than proportionately. Across all delivery methods presented, the older the respondent, the higher the elasticity of demand in absolute terms. We found that WTP was always lower across all respondent characteristics for the online program compared with face-to-face programs having traditional leaders and CHWs. The elasticity of demand for the online program was always consistently greater than 1, regardless of the respondents’ characteristics.

**Table 3:** Willingness to Pay and Elasticity of Demand, by Delivery Method and Responders’ Characteristics, Study of Supply and Demand for Diabetes Prevention Programs in North Carolina, 2015

Characteristic	Traditional		CHW		Online	
	WTP (95% CI)	E_d_ a	WTP (95% CI)	E_d_ a	WTP (95% CI)	E_d_ a
**Sex**						
Male	38 (27 to 49)	0.59	32 (23 to 42)	0.94	14 (7 to 21)	13.08
Female	39 (32 to 46)	0.50	30 (25 to 36)	1.17	21 (17 to 25)	4.46
**Age, y**						
<45	51 (39 to 63)	0.12	38 (28 to 48)	0.55	20 (13 to 28)	4.6
45–54	44 (32 to 56)	0.33	38 (28 to 49)	0.51	27 (19 to 34)	1.93
55–64	34 (24 to 43)	0.80	27 (19 to 35)	1.48	16 (10 to 22)	8.64
≥65	30 (19 to 41)	0.98	22 (12 to 31)	1.94	13 (7 to 20)	15.48
**Race/ethnicity**						
White	29 (20 to 38)	1.02	18 (10 to 25)	2.45	14 (8 to 19)	15.23
African American	44 (37 to 51)	0.33	38 (32 to 43)	0.58	23 (18 to 27)	3.38
**Participated in a structured weight loss program or DPP**						
Yes	40 (31 to 49)	0.51	30 (22 to 38)	1.22	21 (15 to 26)	4.48
No	38 (31 to 45)	0.54	31 (25 to 38)	1.04	18 (13 to 23)	6.66
**Employment status**						
Employed (full-time or part-time)	49 (42 to 56)	0.05	39 (33 to 46)	0.40	24 (20 to 29)	2.71
Unemployed or retired	27 (19 to 35)	1.13	20 (14 to 27)	2.15	13 (8 to 18)	17.86
**Education**						
Some high school	12 (−15 to 38)	1.91	12 (−12 to 36)	3.17	9 (−8 to 27)	82.5
High school diploma	35 (23 to 47)	0.74	36 (26 to 47)	0.68	21 (13 to 28)	4.54
Some college (13–15 y)	33 (24 to 43)	0.71	30 (21 to 38)	1.19	18 (12 to 24)	6.49
College degree (16 y)	48 (39 to 57)	0.13	30 (22 to 38)	1.16	21 (15 to 27)	4.21
Postgraduate (≥17 y)	42 (20 to 64)	0.42	33 (13 to 52)	0.97	13 (−1 to 27)	18.13
**There are not enough affordable weight loss programs in my community**						
Agree	41 (34 to 48)	0.46	31 (25 to 37)	1.14	21 (16 to 25)	4.48
Disagree	34 (24 to 44)	0.65	31 (22 to 39)	1.10	17 (10 to 23)	7.95

Abbreviations: CHW, community health worker; CI, confidence interval; DPP, diabetes prevention program; E_d_, elasticity of demand; WTP, willingness to pay.

a E_d_ measured at price per month = 50. E_d_ presented in absolute values. E_d_ >1 demand is elastic; E_d_ <1 demand is inelastic.

### Provider questionnaire

Each of the 122 local health department and community health center leaders was sent an invitation to complete the survey. Twenty-nine responded; most respondents represented local health departments (27 of 29), and most of these were health directors (Table 1). Respondents represented 27 of the 100 counties in North Carolina.

All respondents rated diabetes as important or very important on their list of health concerns, but less than 60% (17 of 29) had offered CDC’s National Diabetes Prevention Program, an adaptation of the DPP, or some other type of weight-loss program. Of those who had offered DPPs, 2 responding organizations did so as recently as 2015 (Appendix).

**Figure 2:**
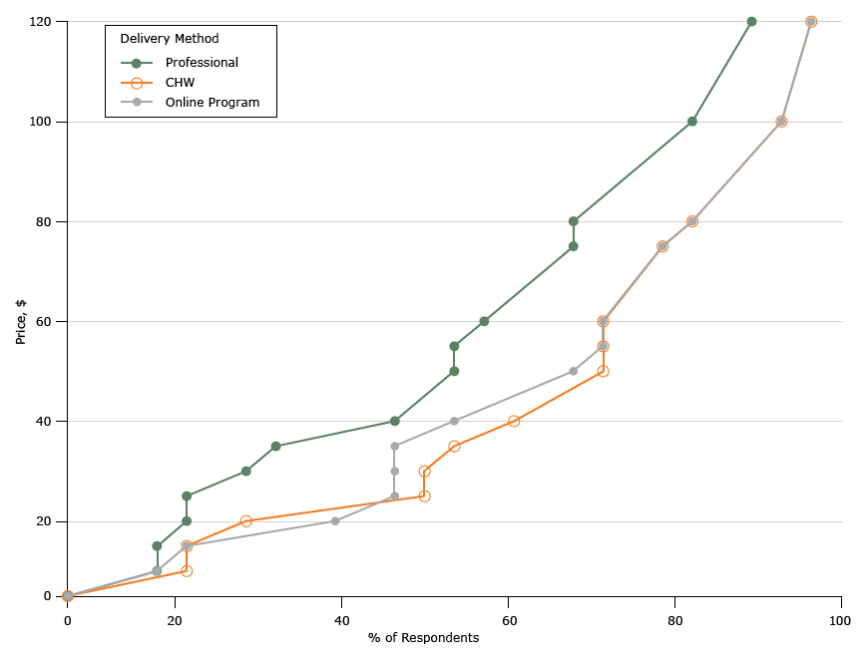
Supply for diabetes prevention programs per month, North Carolina, 2015. Abbreviation: CHW, community health worker. **Table:** 

Among organizations that offered DPP or other weight-loss programs, an average of 1 or 2 staff members had been hired to lead those programs, and up to 250 people enrolled per session. We asked respondents about the minimum price per person per month (equivalent to 4 encounters) at which they would be able to provide the service if the DPP was led by a registered professional, led by a CHW, or delivered online (Table 2). None of the organizations had ever hired a CHW. The reported cost of the professionals hired ranged from $15 per hour for a fitness coach to $38 per hour for a pharmacist, with all other class leaders (health educator, dietitian, and nurse) falling in between. Only 1 local health department reported hosting an online weight-loss program but was unable to provide a breakdown of program costs. To generate a supply schedule for each delivery method, we rank-ordered the respondents’ answers and computed the fraction of providers who would have delivered the program to the community at the different levels of self-reported prices. Figure 2 shows the 3 hypothetical supply schedules. As expected, higher perceived wages for professional leads translated into a smaller fraction of providers willing to supply at each reimbursement level compared with CHWs. The hypothetical supply function for CHW-led and online programs roughly overlap. In Figure 2, we truncated prices to $120 to maintain the same axes as the demand function. However, a small fraction of responders would not provide any services at that level of reimbursement. Table 2 illustrates the highest minimum prices reported ($400 for traditional and online and $200 for a CHW).

Price, $	Delivery Method, % of Respondents		
	Professional	Community Health Worker	Online Program
0	0	0	0
5	7.14	7.14	7.14
10	17.86	21.43	17.86
15	17.86	21.43	21.43
20	21.43	28.57	39.29
25	21.43	50.00	46.43
30	28.57	50.00	46.43
35	32.14	53.57	46.43
40	46.43	60.71	53.57
45	50.00	66.07	60.71
50	53.57	71.43	67.86
55	53.57	71.43	71.43
60	57.14	71.43	71.43
65	60.71	73.81	73.81
70	64.29	76.19	76.19
75	67.86	78.57	78.57
80	67.86	82.14	82.14
85	71.43	84.82	84.82
90	75.00	87.50	87.50
95	78.57	90.18	90.18
100	82.14	92.86	92.86
105	83.93	93.75	93.75
110	86.31	94.94	94.94
115	88.10	95.83	95.83
120	89.29	96.43	96.43

### Cost sharing

By combining WTP estimates with the self-reported cost of providing DPP, we found that classes led by CHWs and registered professionals were the most likely to serve the biggest share of the population (50% and 52%, respectively), whereas online programs were the least likely (33%).

We considered the impact of an arbitrary but reasonable $15 monthly government subsidy (Table 2). The subsidy artificially shifts the consumer’s demand upward and rightward, creating greater demand for the program. Taking the estimated supply curves as given and assuming the same subsidy would be provided to all respondents, instead of paying $44 for the traditional program, $31 for the CHW-led program, and $17 for the online program, respondents would pay out of pocket $37, $25, and $10, respectively, and providers would receive $52, $40, and $25, respectively. Beneficiaries would be better off, because more people would be able to participate while providers would be able to offer the program to more people and receive a higher price in return. A $15 per-respondent subsidy in a state like North Carolina, with 512,000 individuals with prediabetes, would serve 46% to 57% of eligible respondents and cost $3.5 to $4.3 million.

## Discussion

Contingent valuation has become widespread in the evaluation of health interventions (8–14). We provided separate measures of average WTP and price elasticity of demand by delivery model and respondent type, and we estimated the cost of providing the DPP under different delivery models. We also estimated possible DPP uptake in the presence of a government subsidy. Elasticities play an important role in determining how the subsidy is shared among beneficiaries and providers. The more elastic the supply curve (CHW and online supply vs professional-led mode of delivery), the lower the subsidy received by the provider and the greater the transfer to the user. In contrast, the greater the elasticity of demand, the lower the subsidy transfer to the beneficiary. Online programs and CHWs, as well as subsidies, may therefore help scale up DPPs.

This study has limitations. First, the sample sizes in the demand and provider questionnaires were small. We therefore did not randomize the order in which payment amounts were presented to responders; for each, we started with the median price ($50). However, if the starting value in the question is well above the respondent’s true WTP, the respondent will increase the stated WTP amount. The framing effect resulted in a kink at that price. The sequence in which each delivery model was presented to respondents, however, varied randomly to avoid order effects across programs. Our small convenience sample consisted of predominantly medium- and low-income African American women in their 50s, which may have consequences for external validity. For example, a study in a sample of mostly white veterans found higher adherence and satisfaction for the online delivery model compared with the in-person sessions (24).

Second, general concerns about the validity of contingent valuation as a way of eliciting WTP (25,26) could be that respondents may give higher values in a hypothetical situation than what they would pay in a real situation or may give different WTP amounts, depending on the payment method announced (eg, up front, monthly). Furthermore, respondents may purposefully provide a false answer to influence a particular outcome. For example, they may strategically say that they wish to pay zero in the hopes that the program will be offered at no charge. We included the following question in the questionnaire to single out protestors: “Suppose that the program is free, would you enroll?” However, no respondents answered no.

More work is needed to capture more information on respondent types. In the future, salient demographic characteristics should be accounted for to evaluate the effect of incentives such as monetary incentives, child care, and transportation transfers geared to increase uptake in particular subgroups. Despite these limitations, our results were not statistically different from those of Johnson et al (27) (WTP of $42 per month) or those of Jerome et al (28) (WTP of $45 per month).

The success of the DPP depends on the population’s willingness to enroll and complete the program. The policy interventions that could be implemented should be geared at shifting such demand function outward. The ideal subsidy should be set at the breakeven point, where savings from the program (eg, possible reduction in health care use) offset program costs.

## Appendix Supplementary Material to Feasibility Study of Supply and Demand for Diabetes Prevention Programs in North Carolina

This appendix is available for download as a Microsoft Word document.

## References

[1] CDC national diabetes fact sheet. Atlanta (GA): Centers for Disease Control and Prevention, National Center for Chronic Disease Prevention and Health Promotion; 2016. http://www.cdc.gov/chronicdisease/resources/publications/aag/pdf/2016/diabetes-aag.pdf. Accessed September 28, 2016.

[2] Knowler WC , Barrett-Connor E , Fowler SE , Hamman RF , Lachin JM , Walker EA , ; Diabetes Prevention Program Research Group. Reduction in the incidence of type 2 diabetes with lifestyle intervention or metformin. N Engl J Med 2002;346(6):393–403. 10.1056/NEJMoa012512 11832527 PMC1370926

[3] Tabak AG , Herder C , Rathmann W , Brunner E , Kivimäki M . Prediabetes: a high-risk state for developing diabetes. Lancet 2012;379(9833):2279–90. 10.1016/S0140-6736(12)60283-9 22683128 PMC3891203

[4] Huang Y , Cai X , Mai W , Li M , Hu Y . Association between prediabetes and risk of cardiovascular disease and all cause mortality: systematic review and meta-analysis. BMJ 2016;355:i5953. 10.1136/bmj.i5953 27881363 PMC5121106

[5] 2013 BRFSS body mass index grouping. Have you ever been told by a doctor or other health professional that you have pre-diabetes or borderline diabetes? Raleigh (NC): North Carolina State Center for Health Statistics; 2014. http://www.schs.state.nc.us/data/brfss/2013/nc/nccr/topics.htm#ds. Accessed March 14, 2016.

[6] Knowler WC , Fowler SE , Hamman RF , Christophi CA , Hoffman HJ , Brenneman AT , ; Diabetes Prevention Program Research Group. 10-year follow-up of diabetes incidence and weight loss in the Diabetes Prevention Program Outcomes Study. Lancet 2009;374(9702):1677–86. 10.1016/S0140-6736(09)61457-4 19878986 PMC3135022

[7] Alva ML , Hoerger TJ , Jeyaraman R , Amico P , Rojas-Smith L . Impact Of The YMCA Of The USA Diabetes Prevention Program On Medicare Spending And Utilization. Health Aff (Millwood) 2017;36(3):417–24. 10.1377/hlthaff.2016.1307 28264942

[8] Garfield SA , Malozowski S , Chin MH , Narayan KM , Glasgow RE , Green LW , ; Diabetes Mellitus Interagency Coordinating Committee (DIMCC) Translation Conference Working Group. Considerations for diabetes translational research in real-world settings. Diabetes Care 2003;26(9):2670–4. 10.2337/diacare.26.9.2670 12941736

[9] Registry of recognized organizations. Atlanta (GA): Centers for Disease Control and Prevention, National Diabetes Prevention Program. https://nccd.cdc.gov/DDT_DPRP/Registry.aspx?STATE=NC. Accessed October 14, 2016.

[10] Spitalnic P. Certification of Medicare Diabetes Prevention Program [letter from Office of the Actuary]. Baltimore (MD): Centers for Medicare and Medicaid Services; March 14, 2016.

[11] Drummond MF , Sculpher MJ , Torrance GW , O’Brien BJ , Stoddart GL . Contingent valuation studies. In: Drummond MF, Sculpher MJ, Torrance GW, O’Brien BJ, Stoddart GL, editors. Methods for the economic evaluation of health care programs. 3rd edition. Oxford (UK): Oxford University Press; 2005.

[12] Diener A , O’Brien B , Gafni A . Health care contingent valuation studies: a review and classification of the literature. Health Econ 1998;7(4):313–26. 10.1002/(SICI)1099-1050(199806)7:4<313::AID-HEC350>3.0.CO;2-B 9683092

[13] Blumenschein K , Johannesson M . Use of contingent valuation to place a monetary value on pharmacy services: an overview and review of the literature. Clin Ther 1999;21(8):1402–17, discussion 1401. 10.1016/S0149-2918(99)80041-1 10485511

[14] Lin PJ , Cangelosi MJ , Lee DW , Neumann PJ . Willingness to pay for diagnostic technologies: a review of the contingent valuation literature. Value Health 2013;16(5):797–805. 10.1016/j.jval.2013.04.005 23947973

[15] Olsen JA , Smith RD . Theory versus practice: a review of ‘willingness-to-pay’ in health and health care. Health Econ 2001;10(1):39–52. 10.1002/1099-1050(200101)10:1<39::AID-HEC563>3.0.CO;2-E 11180568

[16] Tarasiuk A , Simon T , Regev U , Reuveni H . Willingness to pay for polysomnography in children with obstructive sleep apnea syndrome: a cost-benefit analysis. Sleep 2003;26(8):1016–21. 14746384 10.1093/sleep/26.8.1016

[17] Goswami ND , Hecker E , Holland DP , Naggie S , Cox GM , Mosher A , Feasibility and willingness-to-pay for integrated community-based tuberculosis testing. BMC Infect Dis 2011;11(1):305. 10.1186/1471-2334-11-305 22047015 PMC3217890

[18] Pedersen LB , Gyrd-Hansen D , Kjær T . The influence of information and private versus public provision on preferences for screening for prostate cancer: a willingness-to-pay study. Health Policy 2011;101(3):277–89. 10.1016/j.healthpol.2011.05.008 21680041

[19] Centers for Disease Control and Prevention, National Diabetes Prevention Program. CDC prediabetes screening test. http://www.cdc.gov/diabetes/prevention/pdf/prediabetestest.pdf. Accessed October 5, 2016.

[20] Keyserling TC , Samuel-Hodge CD , Pitts SJ , Garcia BA , Johnston LF , Gizlice Z , A community-based lifestyle and weight loss intervention promoting a Mediterranean-style diet pattern evaluated in the stroke belt of North Carolina: the Heart Healthy Lenoir Project. BMC Public Health 2016;16(1):732. 10.1186/s12889-016-3370-9 27495295 PMC4975883

[21] Hanneman M , Loomis J , Kanninen B . Statistical efficiency of double bounded dichotomous choice valuation. Am J Agric Econ 1991;73(4):1255–63. 10.2307/1242453

[22] López-Feldman A. Introduction to contingent valuation using Stata. Munich Personal RePEc Archive; 2012. MPRA paper no. 41018.

[23] Friedman DD . The firm. In: Friedman DD, editor. Price theory: an intermediate text. Mason (OH): Thomson South-Western Publishing Co; 1985.

[24] Moin T , Ertl K , Schneider J , Vasti E , Makki F , Richardson C , Women veterans’ experience with a web-based diabetes prevention program: a qualitative study to inform future practice. J Med Internet Res 2015;17(5):e127. 10.2196/jmir.4332 26006697 PMC4468391

[25] Carson RT . Contingent valuation: a practical alternative when prices aren’t available. J Econ Perspect 2012;26(4):27–42. 10.1257/jep.26.4.27

[26] Hausman J . Contingent valuation: from dubious to hopeless. J Econ Perspect 2012;26(4):43–56. 10.1257/jep.26.4.43

[27] Johnson FR , Manjunath R , Mansfield CA , Clayton LJ , Hoerger TJ , Zhang P . High-risk individuals’ willingness to pay for diabetes risk-reduction programs. Diabetes Care 2006;29(6):1351–6. 10.2337/dc05-2221 16732020

[28] Jerome GJ , Alavi R , Daumit GL , Wang NY , Durkin N , Yeh HC , Willingness to pay for continued delivery of a lifestyle-based weight loss program: the Hopkins POWER trial. Obesity (Silver Spring) 2015;23(2):282–5. 10.1002/oby.20981 25557807 PMC4310798

