# Supplementary material for: A Feasibility Study of Supply and Demand for Diabetes Prevention Programs in North Carolina
Source: Prev Chronic Dis. 2017 Jun 29;14:E51. doi: 10.5888/pcd14.160604 (PMC5494814; doi:10.5888/pcd14.160604)
Supplement: Supplementary file 1 [file 16_0604Appendix.docx]

# Appendix

## A.1 Summary of the Payments Made on Behalf of Participants in the Considered Nationwide DPP Expansion by the Office of the Actuary Analysis
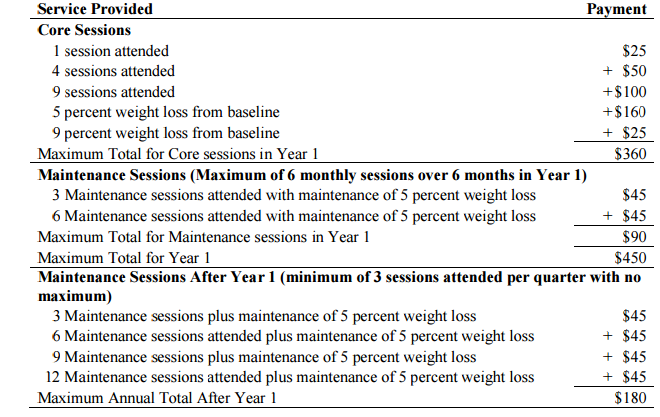


Source: <<https://www.cms.gov/Research-Statistics-Data-and-Systems/Research/ActuarialStudies/Downloads/Diabetes-Prevention-Certification-2016-03-14.pdf>>

## A.2 WTP Questionnaire Tree: Are You Willing to Pay $ Monthly for the Delivery Mechanism Described?


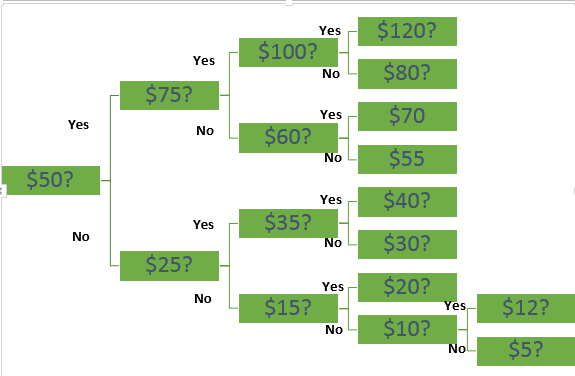


If the responder answers yes to the first question (s)he is asked about his willingness to pay for a higher amount. If (s)he answers no to the first question, a lower amount is offered.

In this setup, each respondent provides the following information set:

1. If the individual answers yes to the first question and no to the second, then t2 > t1. In this case, we can infer that t1 ≤ WTP < t2.

Pr(y,n)=Pr(bid1$\leq x\beta+u<$bid2)=$\Phi\left( x\frac{\beta}{\sigma}-\frac{bid1}{\sigma} \right)-\Phi\left( x\frac{\beta}{\sigma}-\frac{bid2}{\sigma} \right)$

2. If the individual answers yes to the first question and yes to the second, then t2 ≤ WTP < ∞.

Pr(y,y)=Pr($x\beta+u$>bid1, $x\beta+u\geq$bid2)=$\Phi\left( x\frac{\beta}{\sigma}-\frac{bid2}{\sigma} \right)$

3. If the individual answers no to the first question and yes to the second, then t2 < t1. In this case, we have that t2 ≤ WTP < t1.

Pr(n,y)=Pr(bid2$\leq x\beta+u<$bid1)=$\Phi\left( x\frac{\beta}{\sigma}-\frac{bid2}{\sigma} \right)-\Phi\left( x\frac{\beta}{\sigma}-\frac{bid1}{\sigma} \right)$

4. If the individual answers no to the first and second questions, then 0 < WTP < t2.

Pr(n,n)=Pr($x\beta+u<$bid1, $x\beta+u$ $\leq$bid2)=$1-\Phi\left( x\frac{\beta}{\sigma}-\frac{bid2}{\sigma} \right)$

A.3 Type of Programs Offered by Providers Who Responded to Our Questionnaire

|  |  | Any other weight loss program | |  |
| --- | --- | --- | --- | --- |
| Program | Offered? | No | Yes | Total |
| DPP | No | 12 | 9 | 21 |
|  | Yes | 5 | 3 | 8 |
|  | Total | 17 | 12 | 29 |

Note: DPP = Diabetes Prevention Program

## A.4 Alternative Supply Elicitation Question

Suppose your potential funding source (e.g. NC Division of Public Health) sets a flat rate reimbursement rate of:

| Total Amount $ per Course (16 sessions) | Would you chose to provide the service? (yes/no/don’t know) | How many people would you be able to enroll at this level of founding? (min-max range) |
| --- | --- | --- |
| 1,000 | 6/17/5 | 2–60 |
| 2,000 | 7/16/5 | 4–60 |
| 5,000 | 15/8/5 | 8–100 |
| 7,000 | 18/4/6 | 10–140 |
| 10,000 | 22/0/6 | 10–500 |

Note: Only 28 individuals answered this section. One person left all fields blank; blank fields could be interpreted as “not known.”
